# Assessing the Impact of Person-Environment fit on Turnover Intention Through Multiple Linear Regression

**DOI:** 10.1177/00332941231219957

**Published:** 2023-12-12

**Authors:** Kleinjan Redelinghuys

**Affiliations:** Department of Industrial Psychology and People Management, 61799 University of Johannesburg, Auckland Park, South Africa

**Keywords:** Multiple linear regression, demands-abilities fit, needs-supplies fit, person-environment fit, person-organization fit, turnover intention

## Abstract

Focusing on key antecedents of intended turnover may enable organizations to intervene before reaching scenarios where employee turnover is inevitable. The current study aimed to establish which type of fit plays the most prominent role in employees’ turnover intention. To achieve this aim, multiple linear regression was used. By establishing which type of fit influences employees’ thoughts of quitting the most, organizations can adopt better retention strategies as opposed to casting a net and hoping for the best. This cross-sectional study used secondary data from 384 retail employees. Three distinct fit scales and a turnover intention scale were administered. The study’s hypothesis was assessed through multiple linear regression, relative weight analysis, and commonality analysis. Needs-supplies fit was the only variable that had a statistically significant negative association with turnover intention. Needs-supplies fit had the biggest contribution (23.64%) to the multiple *R*^*2*^ value (34.25%) of the multiple linear regression model. Commonality analysis showed that needs-supplies fit accounted for the highest percentage (16.85%) of unique variance in explaining turnover intention. To enhance the probability of retaining employees, organizations may want to fine-tune their retention strategies based on establishing congruence between what employees need and what can be done to meet these desired needs. The current study stresses the importance of distinguishing between different types of person-environment fit as they may not similarly relate to outcomes.

## Introduction

Empirically, employee turnover is often the aftermath of turnover intention (e.g., Hom et al., 2017). The aforementioned can be difficult to navigate as it may either “be part of a natural and healthy attrition that benefits an organization, or it can be problematic or even devastating when companies lose critical talent” (Ju & Li, 2019, p. 214). Although the strength of the association between actual turnover and turnover intention has been questioned (e.g., Rubenstein et al., 2018), Lazzari et al. (2022) argue that focusing on key antecedents of intended turnover may enable organizations to intervene before reaching scenarios where employee turnover is inevitable. Some of these key antecedents include job satisfaction, burnout, job stress, trust, perceived organizational support, organizational commitment, professional identification, intrinsic motivation, work engagement, and justice (Jolly et al., 2021; Li & Yao, 2022; Park & Min, 2020). Another antecedent that regularly plays an integral part in employees’ thoughts of quitting, is person-environment fit. This may be ascribed to person-environment fit’s ability to exert a direct influence on turnover intention, or indirectly through variables such as burnout and job satisfaction (Andela & van der Doef, 2019). Consequently, turnover intention was studied from the lens of person-environment fit in the current study.

Person-environment fit research is often critiqued for not being assessed from a multidimensional perspective, as some argue that “focusing on only one or a few types of fit generates a limited picture of the effects of fit” (Chuang et al., 2016, p. 68). This may be ascribed to the unique effects different subdimensions of person-environment fit may have on employee and organizational outcomes (Ostroff et al., 2005). For example, research has shown that some types of person-environment may be more relevant than others when examining job attitudes and turnover intention (e.g., Kristof-Brown et al., 2005). Therefore, although single-scale person-environment fit studies may assist us in determining the relational strength between person-organization fit and turnover intention, for example, it hinders us from assessing the importance of person-organization fit in influencing turnover intention relative to that of other potentially relevant types of person-environment fit (e.g., person-job fit).

Furthermore, studies have shown that person-environment fit as a second-order factor model significantly associates (directly or indirectly) with turnover intention. For example, Redelinghuys and Botha (2016) showed that person-environment fit indirectly affected turnover intention through job satisfaction. Janse van Rensburg et al. (2017) also found that person-environment fit indirectly affected turnover intention, although through flourishing at work. In contrast, Redelinghuys et al. (2019) found that person-environment fit did not indirectly affect turnover intention through flourishing at work, but rather established a significant direct relationship between person-environment fit and turnover intention. Although the preceding studies provide valuable insights on how the global assessment of person-environment fit associate with turnover intention, they offer little information on whether specific types of fit play a larger role than others in explaining the variance in turnover intention. By establishing which type of fit influences employees’ thoughts of quitting the most, organizations can adopt better retention strategies as opposed to casting a net and hoping for the best. Given the preceding discussion, the current study aimed to establish which type of fit plays the most prominent role in employees’ turnover intention. To achieve this aim, multiple linear regression was used.

### Person-Environment Fit and Turnover Intention

Due to widespread disagreement about how *person-environment fit* should be conceptualized and measured, conceptual clarity is needed when alluding to different aspects of person-environment fit (Yang et al., 2008). The current study used Cable and DeRue’s (2002) conceptualization of three distinct types of fit: person-organization fit, needs-supplies fit, and demands-abilities fit. *Person-organization fit* indicates perceived value similarity between organizations and employees (Cable & DeRue, 2002). *Needs-supplies fit* consider the extent to which employees perceive that their needs (i.e., what they desire from their job) are being met by the supplies (i.e., monetary or non-monetary rewards) offered by organizations (Cable & DeRue, 2002). Lastly, *demands-abilities fit* denotes the perceived match between job demands and employees’ capacity to deal with these demands (Cable & DeRue, 2002). Studies generally find that perceived congruence with different aspects of one’s work environment results in a decrease in turnover intention (e.g., Kristof-Brown et al., 2005). *Turnover intention* relates to employees’ thoughts of terminating their current employment contract and deliberate attempts to secure employment elsewhere (Mobley et al., 1978; Tett & Meyer, 1993).

In line with their hypothesis, Lauver and Kristof-Brown (2001) found person-organization fit to be a stronger predictor of turnover intention than person-job fit. In contrast, Andela and van der Doef (2019) and findings from Kristof-Brown et al.’s (2005) meta-analysis showed the opposite. Furthermore, Kristof-Brown et al. (2005) showed that needs-supplies fit may play a larger role than demands-abilities fit in influencing turnover intention and that it may be useful to distinguish between the two. This notion is supported by other studies (e.g., Abdalla et al., 2018; Morrow & Brough, 2019), where needs-supplies fit significantly associated with turnover intention, whereas demands-abilities fit did not. Some have also argued that it might be easier to cope with demands-abilities misfit than needs-supplies misfit (Van Vianen, 2018). Like Lauver & Kristof-Brown, (2001), Abdalla et al. (2018) found that person-organization fit, as assessed by value congruence, had a larger association with turnover intention than needs-supplies fit, although both were statistically significant. In contrast to Abdalla et al. (2018), Scroggins (2007), as well as Morrow and Brough (2019) however found that needs-supplies fit significantly associated with turnover intention, whereas person-organization fit did not. From the types of perceived fit, person-organization fit and needs-supplies fit appear to affect turnover intention significantly, whereas demands-abilities fit seems to have a minimal effect. Although both person-organization fit and needs-supplies fit generally associate with turnover intention, mixed findings make it hard to establish which of the two types of fit may influence turnover intention the most. To guide this decision, statistical and theoretical arguments are made.

From a statistical standpoint, in simple regression, the proportion of explained variance equals *r*^2^. Based on this principle, the type of fit that correlates the highest with turnover intention should explain the most variance in the latter. Several meta-analytic studies (Kristof-Brown et al., 2005; Oh et al., 2014) have shown that compared to person-organization fit, person-job fit has a stronger average correlation with turnover intention. Based on this study’s conceptualization and measurement of person-environment fit (Cable & DeRue, 2002), others (e.g., Maloba & Pillay-Naidoo, 2022; Morrow & Brough, 2019; Redelinghuys et al., 2019) have also found that from the three types of fit, needs-supplies and turnover intention has the strongest correlation. Hence, needs-supplies fit should potentially affect turnover intention the most. Although this makes sense statistically, it should also make sense theoretically. However, before delving into the needs-supplies fit – turnover intention relationship, it may be useful to explain needs-supplies fit in greater depth.

It is important to note that needs-supplies fit expands beyond monetary gain, as it also includes a wide range of non-monetary rewards (Cable & DeRue, 2002). Although money is generally a good existential motivator, some studies have shown that non-monetary rewards can be equally effective and have a longer-lasting impact on employees than monetary rewards (e.g., Rybnicek et al., 2019). Hence, the different circumstances employees find themselves in will largely dictate which factors will play the most prominent role in their needs-supplies fit perceptions. Among others, some of these circumstances may relate to one’s age, career stage, marital status, and parental status. For example, younger workers with no family commitments may find higher remuneration as opposed to comprehensive benefits more appealing, whereas older employees with family commitments may find the latter more appealing (Cable & DeRue, 2002). Others may experience additional or entirely different needs, like a need for training, respectful leadership, gamified elements to their work, autonomy, relatedness, competence, empowerment, achievement, power, interesting and stimulating work, recognition, flexible working arrangements, work-life balance, meaningful work, and favorable working conditions, to name a few (e.g., Cable & DeRue, 2002; Deci & Ryan, 1985; Herzberg et al., 1959; Koo et al., 2020; McClelland, 1961; Mitchell et al., 2020; Rybnicek et al., 2019; Yeoman, 2014). Consequently, due to the uniqueness of employees and their situation, the importance they attach to different needs and how they respond to unmet needs may vastly differ.

In relation to turnover intention, Cable and DeRue (2002) suggest that needs-supplies fit reflects the extent to which employees are attached to their job or profession, rather than their organization. This may be ascribed to the belief that well-fitting employees from a needs-supplies fit perspective may receive similar benefits at a different organization, as perceived congruence stems from their job, and not their organization (Cable & DeRue, 2002). As there is not a fixed set of needs and accompanying supplies in person-environment fit research, employees may among others experience monetary, psychological, and/or social needs (Cable & DeRue, 2002). In relation to this non-exhaustive list of needs, an extensive review (Hom et al., 2017) of employee turnover research and meta-analytic findings (e.g., Griffeth et al., 2000; Li & Yao, 2022; Park & Min, 2020) have shown that turnover intention or turnover associate with autonomy, benefits, met expectations, pay satisfaction, rewards, and training; each of which could be indicative of needs-supplies fit. Consequently, the following hypothesis is formulated:

Hypothesis 1 Of the three distinct types of fit, needs-supplies fit influences turnover intention the most.

## Method

### Participants

In total, 398 employees were sampled from a South African retail firm, although 14 observations were deleted due to missing data. The remaining 384 employees were predominantly black (*n* = 218, 56.8%) female (*n* = 267, 69.5%) employees between the ages of 31 and 50 (*n* = 300, 78.2%). Upwards of 50% of employees were employed for a period between one and five years (*n* = 203, 52.9%). The majority of employees listed a Grade 12 certificate as their highest level of education (*n* = 234, 60.9%). Regarding job level, most participants did not occupy supervisory or managerial positions (*n* = 338, 88%).

### Measuring Instruments

Three distinct measures (Cable & DeRue, 2002) were used to tap into perceived *person-organization fit, needs-supplies fit, and demands-abilities fit*. Each measure contains three items, scored on a 7-point scale (1 = *strongly disagree*, 7 = *strongly agree*). Sample items include: ‘My personal values match my organization’s values and culture’ (person-organization fit) and ‘My personal abilities and education provide a good match with the demands that my job places on me’ (demands-abilities fit). Needs-supplies fit was measured with the following items: ‘The attributes that I look for in a job are fulfilled very well by my present job’, ‘The job that I currently hold gives me just about everything that I want from a job’, and ‘There is a good fit between what my job offers me and what I am looking for in a job’. Confirmatory factor analysis by Hinkle and Choi (2009) showed support for the three types of fit, although better fit was achieved when one of the items of demands-abilities fit was allowed to cross-load onto needs-supplies fit. Maloba and Pillay-Naidoo (2022) found Cronbach’s alpha coefficients ranging from .88 to .93.

The 3-item turnover intention scale (Sjöberg & Sverke, 2000) was used to measure *turnover intention*. It is scored on a 5-point scale (1 = *strongly disagree*, 5 = *strongly agree*). A sample item is ‘If I was completely free to choose I would leave this job’. Redelinghuys et al. (2019) established a reliability coefficient of .91 for the scale.

### Procedure

The researcher collected the primary cross-sectional data in June 2014. The primary data were read into Excel, anonymized, and kept on a password-protected computer, as well as secure cloud storage. The original paper-based questionnaires were destroyed after five years. The secondary data was used in this study to investigate a different hypothesis from those of other studies that utilized the dataset (see Redelinghuys & Botha, 2016; Redelinghuys et al., 2020). Further information about the initial research procedure can be found in Redelinghuys and Botha (2016).

### Ethical Considerations

Redelinghuys and Botha (2016) received ethical clearance to collect primary data (Ref: OPT-2014-001). A formal ethics application was submitted to the Research Ethics Committee of the Department of Industrial Psychology and People Management, whereby ethical clearance (Ref: IPPM-2023-723) was granted to use the secondary data. During the primary data collection process, participants received an information sheet that outlined the purpose of the study and the requirements for participation. Participants who were willing to participate were required to sign a consent form and only those who indicated that their data may be used for future research purposes were included in the secondary data analysis.

### Data Analysis

R version 4.2.1 (R Core Team, 2022) was used for all the analyses within the RStudio (RStudio Team, 2022) integrated development environment. The following packages were used: *car* (Fox & Weisberg, 2019), *Hmisc* (Harrell, 2022), *lavaan* (Rosseel, 2012), *lm.beta* (Behrendt, 2022), *lmtest* (Zeileis & Hothorn, 2002), *MBESS* (Kelley, 2022), *olsrr* (Hebbali, 2020), *psych* (Revelle, 2022), *psychometric* (Fletcher, 2022), *relaimpo* (Grömping, 2006), *rwa* (Chan, 2020), *semTools* (Jorgensen et al., 2022), *stats* (R Core Team, 2022), and *yhat* (Nimon et al., 2021). Detailed information about the analyses, packages employed, and statistical cut-off points used in the current study are elaborated on in the results section for ease of interpretability.

## Results

### Screening

Before analyzing the data, it was screened for duplicated and missing values, as well as possible data entry errors. Using the *duplicated* function in *base R*, no duplications were found in the full dataset. This was determined by excluding the ‘ID’ variable and assessing if any participant’s response identically overlapped with another regarding their biographical information and their responses to the person-organization fit, needs-supplies fit, demands-abilities fit, and turnover intention items. In total, 16 missing data points were established: person-organization fit (*n* = 2), needs-supplies fit (*n* = 7), demands-abilities fit (*n* = 6), and turnover intention (*n* = 1). Observations that contained missing values on the variables of concern, were discarded through listwise deletion, which resulted in approximately 3.5% data loss (14 observations less than the original sample). Although many strongly advise against listwise deletion, the percentage of missing data was considered small enough not to substantially affect the results of the study. No data entry errors were detected for any of the constructs’ items as all the reported values fell within the minimum and maximum ranges as per each questionnaire’s response format.

### Descriptive Statistics, Reliability Coefficients, and Correlation Coefficients

Descriptive statistics were calculated by the *describe* function from the *psych* (Revelle, 2022) package. Reliability coefficients (α and ω) were established through the *ci.reliability* function in the *MBESS* (Kelley, 2022) package. Lastly, Pearson correlation coefficients were calculated by the *rcorr* function in the *Hmisc* (Harrell, 2022) package. Table 1 reports the results.

**Table 1.** Descriptive Statistics, Reliability Coefficients, and Correlation Coefficients.

	*M*	*SD*	Skewness	Kurtosis	α	ω	1	2	3
1 Person-organization fit	4.62	1.50	−.62	−.39	.85	.85			
2 Needs-supplies fit	4.39	1.65	−.29	−.93	.88	.88	.56*		
3 Demands-abilities fit	4.94	1.51	−.87	.01	.85	.85	.49*	.66*	
4 Turnover intention	2.89	1.27	.08	−1.09	.90	.90	−.34*	−.59*	−.38*

*Note. M =* Mean; *SD =* Standard deviation; α = Cronbach’s alpha reliability coefficient; ω = McDonald’s coefficient omega. *Statistically significant (*p* < .01).

Table 1 illustrates that all the correlation coefficients between the constructs are statistically significant (*p* < .01). Reliability coefficients exceeding .80, as observed in Table 1, are generally deemed acceptable for research purposes (Nunnally, 1978).

### Validity

Prior studies have given a fair amount of attention to the validity of person-organization fit, needs-supplies fit, and demands-abilities fit (Cable & DeRue, 2002; Hinkle & Choi, 2009). Confirmatory factor analysis was conducted to test four feasible measurement models in congruence with Cable and DeRue’s (2002) study. These models were carefully chosen by Cable and DeRue (2002) to determine how person-organization fit, needs-supplies fit, and demands-abilities fit are best modeled based on different theoretical frameworks of person-environment fit. In Model 1, three first-order factors were specified: person-organization fit, needs-supplies fit, and demands-abilities fit (three items each). In Model 2, one first-order factor was specified that captured an overall perception of fit (nine items). In Model 3, two first-order factors were specified: supplementary fit (three items of person-organization fit) and complementary (combining three items of needs-supplies fit and three items of demands-abilities fit). In Model 4, two first-order factors were specified: value congruence (combining three items of person-organization fit and three items of needs-supplies fit) and demands-abilities fit (three items). Each model’s performance was assessed through commonly reported goodness-of-fit statistics (see Table 2). Values close to .95 (comparative fit index and Tucker-Lewis index), .06 (root mean square error of approximation), and .08 (standardized root mean square residual) served as markers of adequate model fit (Hu & Bentler, 1999). Furthermore, lower values in terms of the Akaike information criterion and the Bayesian information criterion are desirable. All the models were tested with the *cfa* function in the *lavaan* (Rosseel, 2012) package. The MLR estimator was used in this regard. Table 2 presents the results.

**Table 2.** Competing Measurement Models for Person-Organization Fit, Needs-Supplies Fit, and Demands-Abilities Fit.

Model	𝜒^2^	*df*	𝜒^2^/*df*	TLI	*CFI*	RMSEA estimate [90% CI]	SRMR	AIC	BIC
1	53.743	24	2.24	.966	.977	.057 [.04, .07]	.04	11667.239	11750.203
2	333.289	27	12.34	.684	.763	.172 [.16, .19]	.10	12110.453	12181.565
3	160.327	26	6.17	.856	.896	.116 [.10, .13]	.06	11829.437	11904.499
4	245.285	26	9.43	.765	.831	.148 [.14, .16]	.09	11958.027	12033.089

*Note.* χ^2^ = chi-square statistic; *df* = degrees of freedom; TLI = Tucker-Lewis index; CFI = comparative fit index; RMSEA = root mean square error of approximation with 90% confidence intervals; SRMR = standardized root mean square residual; AIC = Akaike information criterion; BIC = Bayes information criterion.

Table 2 indicates that Model 1 fitted the data the best. For this model, all the relevant goodness-of-fit statistics fell within acceptable ranges. This coincided with Cable and DeRue’s (2002) findings that it is appropriate to model person-organization fit, needs-supplies fit, and demands-abilities fit as three distinct types of perceived fit.

Due to the high correlations between person-organization fit, needs-supplies fit, and demands-abilities fit, which ranged from .49 to .66 (see Table 1), it was deemed important to assess the discriminant validity between the constructs using the heterotrait-monotrait ratio (HTMT). The *htmt* function from the *semTools* package was used for this purpose. The following values were established: person-organization fit and needs-supplies fit (.64), needs-supplies fit and demands-abilities fit (.76), and person-organization fit and demands-abilities fit (.58). Although precise thresholds are debatable (Henseler et al., 2015), HTMT values <.85 are believed to indicate sufficient discriminant validity. Hence, it seemed reasonable to separate the different types of fit as studies have shown that they are distinct in what they measure, how people perceive them (Cable & DeRue, 2002), and how they relate to various outcomes (e.g., Abdalla et al., 2018).

### Testing the Assumptions of Multiple Linear Regression

To establish whether multiple linear regression is a suitable approach in assessing the relationship between the independent variables (person-organization fit, needs-supplies fit, demands-abilities fit) and turnover intention, it was useful to test some assumptions first to avoid potentially misleading results (Casson & Farmer, 2014). Several plots and statistical techniques were assessed to provide more information regarding assumptions of linearity, homoscedasticity, normality of residuals, and collinearity, as well as the presence of outliers in the dataset.

Inspection of the partial residual plots provided support for the linearity assumption. As heteroscedasticity commonly occurs in cross-sectional studies (Hoffmann, 2021), it was formally tested with the studentized Breusch-Pagan test (*bptest*) from the *lmtest* (Zeileis & Hothorn, 2002) package. The Breusch-Pagan value was not statistically significant (BP = 5.9416, *df* = 3, *p* = .1145), offering support for the homoscedasticity assumption. Furthermore, a QQ-plot was used to assess whether the residuals were normally distributed. For the most part, observations lay close to the 45-degree line. Hence, there is a fair indication of normally distributed residual values. To formally test the normality assumption, the Kolmogorov-Smirnov test was interpreted, using the *ols_test_normality* function from the *olsrr* package. The Kolmogorov-Smirnov statistic (value = .0274) was not statistically significant (*p* > .05), suggesting that the normality assumption seems to hold.

**Figure 1.**
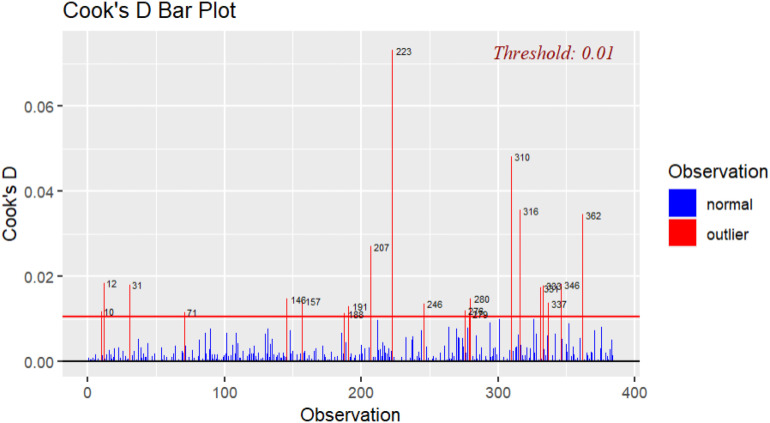
Cook’s D bar plot.

Several plots (e.g., QQ-plot, partial residual plot) that were used for testing assumptions also served the purpose of identifying outliers. As outliers, particularly influential cases, may affect regression results (Stevens, 1984), more tests were conducted to determine the effect of potentially problematic observations. Using the *outlierTest* from the *car* package, results showed that observation 310 had the highest studentized residual value (−3.40). The Bonferroni adjusted *p*-value was however not statistically significant (*p* = .29). The next largest studentized residual values, as determined by the *rstudent* function from the *stats* package, were for observations 223 (−2.89) and 188 (2.79). Potential influential cases were further investigated with the *ols_plot_cooksd_bar* function from the *olsrr* package (see Figure 1).

Figure 1 illustrates that observation 223 had the largest Cook’s distance value in comparison to the remaining values. Rounded off to two digits, a Cook’s distance value of .07 was established using the *cooks.distance* function from the *stats* (R Core Team, 2022) package for observation 223, followed by values of .05 (observation 310) and .04 (observation 316). Observation 223 scored a low score on both needs-supplies fit (*M* = 2), demands-abilities fit (*M* = 1), and turnover intention (*M* = 1). This meant that although their needs were not met by their job and there was a demands-abilities mismatch, they had no intention of quitting. Although this is an unusual phenomenon, it is feasible that employees will remain with their organization when they have low perceived job mobility (e.g., Wheeler et al., 2007), especially in the South African context where unemployment is rife (Williamson & Perumal, 2021)^1^. Furthermore, 21 outliers were identified to breach the .01 threshold. No values however exceeded or equaled 1, which meant that based on the latter (*D* ≥ 1), the values fell within an acceptable range (Hoffmann, 2021). Consequently, no influential observations were deleted.

### Multicollinearity

The *ols_vif_tol* function from the *olsrr* package was used to assess potential multicollinearity in the multiple linear regression model. Kim (2019) suggests that variance inflation factor (VIF) values greater than 5 and tolerance values lower than .20 indicate multicollinearity. The following VIF and tolerance values were established: needs-supplies fit (VIF = 2.03, Tol. = .49), demands-abilities fit (VIF = 1.85; Tol. = .54), and person-organization fit (VIF = 1.51, Tol. = .66). Furthermore, the Condition Index was calculated with the *ols_eigen_cindex* function in the *olsrr* package. A value of 11.09 was established with eigenvalues of 3.86, .06, .05, and .03. Kim (2019) notes that a Condition Index value between 10 and 30 may indicate the existence of multicollinearity, whereas strong evidence of multicollinearity occurs when values exceed 30. Furthermore, Kim (2019) suggests that multicollinearity may become a concern when two or more variance decomposition proportion values higher than .80 accompany condition index values higher than 10. In the current study, variance decomposition values that accompanied the Condition Index (11.09) were: .55, .81, and .05. Hence, results based on the preceding criteria did not provide overwhelming evidence that multicollinearity will have a strong effect on the regression model, although it also did not indicate that it will have no effect.

### Multiple Linear Regression

The multiple linear regression model was specified with the *lm* function from the *stats* package where turnover intention was regressed onto the three independent variables: person-organization fit, needs-supplies fit, and demands-abilities fit. Confidence intervals for the regression coefficients were calculated with the *confint* function from the *stats* package. Standardized regression coefficients were calculated with the *lm.beta* function from the *lm.beta* (Behrendt, 2022) package. R-squared confidence intervals were calculated with the *CI.Rsqlm* function from the *psychometric* (Fletcher, 2022) package. Table 3 presents these results.

**Table 3.** Multiple Linear Regression With the Three Types of Fit as Predictors of Turnover Intention.

Variable	B [95% CI)	β [95% CI]	SE	*t*	*p*	Adjusted *R*^*2*^ [95% CI]	RSE
Intercept	4.87	-	.20	24.24	<.001	.34 [.27, .42]	1.03
Needs-supplies fit	−.45 [−.54, −.36]	−.58 [−.67, −.49]	.05	−9.87	<.001	-	-
Demands-abilities fit	.01 [−.08, .10]	.01 [−.08, .11]	.05	.23	.82	-	-
Person-organization fit	−.01 [−.10, .07]	−.02 [−.10, .07]	.04	−.33	.75	-	-

*Note.* B [95% CI] = unstandardized beta coefficient with 95% confidence intervals; β [95% CI] = standardized beta coefficient with 95% confidence intervals; SE = standard error; Adjusted *R*^*2*^ = Adjusted R-squared with 95% confidence intervals; RSE = residual standard error.

Table 3 indicates that needs-supplies fit was the only variable that had a statistically significant negative association with turnover intention (B = −.45, *p* < .001). The model explained approximately 34% (Multiple *R*^*2*^ *=* 34.25%, Adjusted *R*^*2*^ = 33.73%) of the variance in turnover intention. This suggests a substantial effect size according to Cohen’s (1988) conventional guidelines. The F-statistic (65.98) on 3 and 380 degrees of freedom was statistically significant (*p* < .001). This offers initial support for the study’s hypothesis.

Next, the relative importance of each variable in influencing turnover intention was calculated using the *calc.relimp* function from the *relaimpo* (Grömping, 2006) package to understand the potential effect of multicollinearity. Kalnins (2018) proposes “to mitigate multicollinearity concerns, authors should show separate regressions with only one, then only the other, and then both collinear variables of interest” (p. 2378). Based on this recommendation, the average coefficients for different model sizes are reported in Table 4.

**Table 4.** Unstandardized Beta Coefficients for Different-Sized Linear Regression Models.

	1 independent V	2 independent V’s	3 independent V’s
N-S fit	−.45	−.45	−.45
D-A fit	−.32	−.12	.01
P-O fit	−.28	−.09	−.01

*Note.* The 1, 2, and 3 in front of the Independent V/V’s refer to the number of independent variable/s in the model.

Table 4 shows that each predictor had a notable effect on turnover intention when assessed separately (i.e., simple linear regression models). The coefficients for demands-abilities fit and person-organization fit, however, decreased consistently as more variables entered the regression equation, whereas the coefficients for needs-supplies fit remained stable in each equation (B = −.45). This offers additional support for the study’s hypothesis.

Nimon and Reio (2011) advocate for using a wider range of statistical techniques when it comes to the interpretation of multiple regression, suggesting that the sole interpretation of beta weights is not sufficient. Therefore, further analyses were conducted to see how each variable contributed to the *R*^*2*^ of the multiple linear regression model. Firstly, relative weight analysis was conducted with the *rwa* function in the *rwa* (Chan, 2020) package. Results showed that needs-supplies fit had the biggest contribution (23.64%) to the multiple *R*^*2*^ value (34.25%) of the multiple linear regression model identified earlier, followed by demands-abilities fit (6.09%), and person-organization fit (4.52%). Although relative weight analysis allows one to understand the contribution of variables to the multiple *R*^*2*^ value, it does not make a distinction between common and unique variance. Therefore, to gain a better understanding, commonality analysis was conducted with the *commonalityCoefficients* function from the *yhat* (Nimon et al., 2021) package. Results showed that there were no negative commonality coefficients, which typically imply the absence of suppression (Kraha et al., 2012). Needs-supplies fit’s unique variance contributed 16.85% (49.20% in total) to the multiple *R*^*2*^ value (34.25%) of the multiple linear regression model. The rest of the variance was mostly due to common variance that was shared between the predictor set. The unique contributions of demands-abilities fit (.03% in total) and person-organization fit (.05% in total) were close to zero. Although to differing degrees, both relative weight analysis and common analysis showed that needs-supplies fit explained the most variance in turnover intention compared to the other types of fit. The effect size of the unique variance of needs-supplies fit was moderate according to Cohen’s (1988) conventional guidelines. Therefore, the study’s hypothesis is supported.

### Supplementary Analyses

To assess potential distortions in the study’s results, supplementary analyses were conducted where demographic variables were included as potential confounders. Firstly, a simple regression model was specified using the *lm* function from the *stats* package where turnover intention was regressed onto needs-supplies fit. Next, a multiple regression model was specified where turnover intention was regressed onto needs-supplies fit, person-organization fit, demands-abilities fit, gender, age, race, highest level of education, and job tenure simultaneously. Demographic variables were coded dichotomously: gender (0 = male, 1 = female), age (0 = younger than 30, 1 = older than 30), race (0 = black, 1 = white), highest level of education (0 = Grade 12, 1 = diploma), and job tenure (0 = less than 3 years, 1 = more than 3 years). Categories coded as 0 served as the reference categories. Within several demographic categories, certain groups were discarded due to low representation in comparison to other groups. This included multiracial participants (*n* = 20), Indian participants (*n* = 13), one participant who did not identify with any racial category, as well as participants with a degree (*n* = 14), post-graduate degree (*n* = 14), or technical qualification (*n* = 22). Furthermore, due to low representation in the managerial (*n* = 23) and supervisory (*n* = 21) groups, job level as a potential confounder could not be assessed. Table 5 reports the results.

**Table 5.** Assessing the Impact of Potential Confounding Variables.

Variable	B	SE	t	*p*	Adjusted *R*^2^	RSE
Simple linear regression model						
Intercept	4.97	.17	29.17	<.001	.3735	1.02
Needs-supplies fit	−.47	.04	−13.07	<.001	-	-

Variable	B	SE	t	*p*	Adjusted *R*^2^	RSE
Multiple linear regression model with confounding variables included						
Intercept	4.76	.26	18.60	<.001	.3839	1.01
Person-organization fit	−.00	.05	−.08	.93		
Demands-abilities fit	.03	.05	.54	.59		
Needs-supplies fit	−.48	.05	−9.31	<.001		
Gender (Female)	.25	.14	1.84	.07		
Age (older than 30)	.12	.13	.95	.34		
Race (white)	−.37	.14	−2.68	<.01		
Education (Diploma)	−.14	.14	−.98	.33		
Job tenure (> 3 years)	.07	.13	.53	.59		

*Note.* B = unstandardized beta coefficient; SE = standard error; Adjusted *R*^*2*^ = Adjusted R-squared; RSE = residual standard error. The reference groups were Gender (Male), Age (Younger than 30), Race (Black), Education (Grade 12), and Job tenure (< 3 years).

Table 5 shows that confounding variables had little effect on the initial multiple linear regression model of the study as indicated by a 1.99% change^2^ in the parameter estimate of needs-supplies fit from Model 1 to Model 2, although race also significantly associated with turnover intention to a lesser extent. Similar results were obtained when the confounding variables were assessed individually with the different types of fit and when they were assessed simultaneously with all variables. These results are offered for illustrative rather than interpretive purposes as this did not form part of the study’s hypothesis or analytic strategy. Additional considerations regarding confounding variables are discussed in the limitations section.

## Discussion

The current study aimed to establish which type of fit plays the most prominent role in employees’ turnover intention. To achieve this aim, multiple linear regression was used.

Before comparing the results of the current study to those of previous studies, it is worth noting that the results are not directly comparable due to different statistical methods (e.g., multiple regression, partial least squares analysis, hierarchical regression) being employed, varying sample sizes, and the use of different measuring instruments to capture the study constructs. However, best efforts were made to report contrasting and supporting evidence from fairly similar studies.

Results showed that of the three distinct types of perceived fit, needs-supplies fit had the largest influence on turnover intention. Therefore, needs-supplies fit seems to play a larger role than person-organization fit and demands-abilities fit when it comes to influencing turnover intention. This is consistent with findings from previous studies. For example, Kristof-Brown et al. (2005) found that person-job fit correlated higher with turnover intention than person-organization fit (*r* = −.46 vs. *r* = −.35). They also found that complementary needs-supplies fit correlated higher with turnover intention than person-organization fit (*r* = −.40 vs. *r* = −.38) based on value congruence. Complementary demands-abilities fit had a correlation of −.18 with turnover intention (Kristof-Brown et al., 2005). Furthermore, Scroggins (2007) also found that demands-abilities fit and person-organization fit played a smaller role in predicting turnover intention when assessed in conjunction with other types of person-job fit (e.g., needs-supplies fit, self-concept/job fit). Similarly, Morrow and Brough (2019) found that needs-supplies fit significantly associated with turnover intention, whereas person-organization fit and demands-abilities fit did not have significant associations with the outcome.

Others have, however, found contrasting results. For example, Lauver and Kristof-Brown (2001) found person-organization fit to be a stronger predictor of turnover intention than person-job fit (β = −.47 vs. β = −.22). Similarly, Abdalla et al. (2018) found that person-organization fit, based on value congruence, had a larger association with turnover intention than needs-supplies fit, although both were statistically significant (β = −.18 vs. β = −.11). Furthermore, they found that the association between demands-abilities fit and turnover intention (β = −.03) was not statistically significant. In Lauver and Kristof-Brown’s (2001) case, a potential reason for the contrasting results could be attributed to measuring person-job fit as a unidimensional construct as opposed to making a distinction between needs-supplies fit and demands-abilities fit. As for Abdalla et al. (2018), a potential reason for the contrasting results could be attributed to making alterations to Cable and DeRue’s (2002) perceived fit scale. Abdalla et al. (2018) measured person-organization fit with seven items, needs-supplies fit with five items, and demands-abilities with four items. This deviated from the three items per scale as developed by Cable and DeRue (2002).

Furthermore, results showed that needs-supplies fit, demands-abilities fit, and person-organization fit accounted for approximately 34% of the variance in turnover intention. This is fairly close to the amount of variance explained by previous studies when assessing different types of fit. For example, Andela and van der Doef (2019) found that person-job fit, person-organization fit, person-group fit, and person-supervisor fit explained roughly 35% of the variance in turnover intention. Others have found the following amounts of variance explained: 40.7% (Scroggins, 2007), 30% (Lauver & Kristof-Brown, 2001), and 29% (Morrow & Brough, 2019). Regarding the type of fit that contributed the most to turnover intention, relative weight analysis showed that needs-supplies fit explained the most variance (23.64%), followed by demands-abilities fit, and person-organization fit. In their relative weight analysis, Chuang et al. (2016) found a slightly higher percentage for person-organization fit (7%) compared to person-job fit (6%). Additionally, commonality analysis showed that needs-supplies fit accounted for the highest percentage (16.85%) of unique variance in explaining turnover intention, whereas demands-abilities fit and person-organization fit had little unique variance contributions. Morrow and Brough (2019) found that approximately 4% of the variance explained in turnover intention, was unique to needs-supplies fit.

### Implications

Although the current study could not highlight specific needs that are important in influencing employees’ turnover intention, it could nonetheless determine that needs-supplies fit has a greater impact on the latter than person-organization fit or demands-abilities fit. Therefore, to enhance the probability of retaining employees, organizations may want to fine-tune their retention strategies based on establishing congruence between what employees need and what can be done to meet these desired needs. When greater efforts are made from a management perspective to be attentive to the unique needs of employees, employees should be less likely to think about quitting. Management should however be cautious about making assumptions regarding employee needs (Hills, 2004) and rather gain input from each employee on how their desired fit may be realized in the best way possible. Oversupplying employees with something that they do not need or undersupplying them with something they do need will most likely lead to outcomes that are neither in the employee’s nor the company’s best interests.

Regarding possible implications from a measurement perspective, the amount of common variance that is shared between the predictor set (as per Cable and DeRue’s conceptualization and measurement) and turnover intention may require further investigation. More elaborate factor analytic investigations may provide further insight into the factor structure of the types of fit, and if there is possibly a more appropriate way to model them. Concerns regarding shared variance between different types of fit were also noted in other studies (e.g., Scroggins, 2007). Furthermore, Hinkle and Choi (2009) achieved better model fit when one of the items of demands-abilities fit was allowed to cross-load onto needs-supplies fit.

### Limitations and Recommendations

This study had several limitations, which should be kept in mind when interpreting results. Firstly, due to potential multicollinearity, the exact magnitude of the regression coefficients of the study predictors is somewhat open to interpretation. More clarity regarding the role of each predictor was however provided by supplementing the multiple linear regression analysis with relative weight analysis and commonality analysis, where the variance attributable to each predictor could be evaluated. In line with Nimon and Reio’s (2011) suggestion, we urge future studies to incorporate a wide range of statistical techniques to interpret the contribution of each predictor variable in their multiple regression model. As one can also not make definitive conclusions based on a single study, it may be interesting to see if needs-supplies fit also influences turnover intention the most compared to other types of fit in other samples too.

Secondly, due to not measuring person-environment fit commensurably, polynomial regression and response surface methodology (e.g., Edwards, 2007) could not be applied to each type of fit to ascertain if the addition of nonlinear terms contributes significantly to explaining turnover intention. This is an important consideration, especially for needs-supplies fit, where studies have shown (e.g., Edwards et al., 1998) that a deficiency or excess supplies may either be asymptotic or U-shaped in relation to certain outcomes. Some have also argued “that deficiency tends to be more harmful than excess” (Van Vianen, 2018, p. 91). It may be interesting for future studies to examine these assertions in greater depth.

Thirdly, needs-supplies fit was measured in general terms. Although this helped in stressing the importance of addressing needs-supplies fit, one could not identify how much weight employees assign to one or multiple needs when making perceptions of fit. One could also not identify which needs employees value in general. Future studies may build on prominent need theories (e.g., Deci & Ryan, 1985; McClelland, 1961) to identify and test the importance of different needs for different employees. For example, employee A may experience a need for higher remuneration, power, and achievement, whereas employee B may yearn for additional training, interesting work, and respectful leadership. Qualitative investigations may aid researchers in understanding what goes through an employee’s mind or which factors are weighed up against each other when responding to a statement like ‘There is a good fit between what my job offers me and what I am looking for in a job’.

Fourthly, the effect of confounding variables was assessed in a supplementary way as opposed to being an integral part of the analytic strategy. Future studies are encouraged to incorporate confounding variables from the outset, go into greater depth when assessing their impact, and consider variables other than demographic variables that may act as confounders. For example, in the current study, something like job satisfaction could have also played a role.

Fifthly, although the study assessed different types of person-environment fit, some have argued that person-environment fit extends beyond person-organization fit, needs-supplies fit, and demands-abilities fit. For example, Andela and van der Doef (2019) showed that person-supervisor fit and person-group fit are also associated with turnover intention, although to a lesser extent. Kristof-Brown et al. (2005) reached a similar conclusion regarding person-group fit. In contrast, Abdalla et al. (2018) found person-group fit to be a stronger predictor of turnover intention than person-job fit and person-organization fit. However, in the East Asian context, Oh et al. (2014) found person-supervisor fit to be the most important predictor of turnover intention. To address this inconclusiveness, future studies may want to incorporate a multitude of person-environment fit dimensions simultaneously, while also having a strategy to deal with potential multicollinearity and suppression effects. Cross-cultural and cross-national comparisons between different types of fit may also shed further light on the topic.

Lastly, the exclusive use of self-report measures is often seen as a limitation. From a needs perspective, Rybnicek et al. (2019) suggest that:

While individuals are aware of their conscious needs and are able to express them verbally (e.g. in a questionnaire), they lack access to or awareness of their unconscious needs. Consequently, unconscious needs or motives are difficult to measure and neuroscientific techniques may offer new methods to approach them in a more valid way (p. 472).

## Notes

1. At the time of data collection, the unemployment rate in South Africa was approximately 25.2% (Statistics South Africa, 2014).

2. This was calculated as follows: (−.47321 - −.482605)/–.47321*100. In Table 5 the unstandardized beta coefficients were rounded off to two digits.
